# A Retrospect of Medical Progress, in the State of New York and the County of Erie1Annual address of the President of the Medical Society of the County of Erie, delivered January 12, 1892.

**Published:** 1892-03

**Authors:** E. C. W. O’Brien

**Affiliations:** Buffalo, N.Y.


### A RETROSPECT OF MEDICAL PROGRESS, IN THE STATE OF NEW YORK AND THE COUNTY OF ERIE.[1]

BY E. C. W. O'BRIEN, M. D., Buffalo, N. Y.

ON SUCH an occasion as this it will, I think, not be profitless, if, acting as your representative and speaking in the capacity with which your kindness has invested me, I take a cursory survey of what is going on in our profession at large; and, then, a closer view of what we are doing in our own country, our own State, and our own county. Often as the mirror of medical progress has been appealed to, it is pleasant to take another look. If you compare the medicine and surgery of the present time with the medicine and surgery of even so recent a time as twenty-five years ago, we find on the one hand that method has succeeded to guess-work, and, on the other hand, that the boldness of security has taken the place of the timidity and inaction that are inseparable from ignorance.

In medicine, it is undoubtedly to the study of microörganisms, and their relations to disease—in other words, medical bacteriology—that the advance which everybody recognizes, is largely to be credited. This study, to be sure, has still to be pursued to an extent compared with which its present state is embryonic—perhaps, hardly more than germinal—and by this I mean, not that what we most need is the discovery, and establishment of the

1. Annual address of the President of the Medical Society of the County of Erie, delivered January 12, 1892.

pathogenic relations of more and more microbes, although this, of course, is desirable and sure to be accomplished, but that we have still to learn many times as much as we know, or can reasonably conjecture, at present, as to the ins and outs of the part played by familiar microbes with regard to pathological processes.

But on this subject I need not enlarge; our current literature is full of it. Merely one phase of it—that of the bacteriology of septic and purulent processes—has proved the master factor in the immense strides made by surgery during the closing third of the century. The old surgery had gone about as far as it could go; improvement in technique hardly heightened its efficiency in saving life and limb; something radically new, pervading it throughout, and changing it interstitially, was needed for its regeneration. And that thing came in the form of antisepticism—Listerism, for such it is only right to call it as a trifling tribute to Sir Joseph Lister's great services to surgery and to mankind.

We see their beneficence not only in the better showing made in surgical practice, but also in the impetus to bacteriological study, in its application to general medicine, that might otherwise have required many years for its initiation. To this impetus we owe it that every well-appointed medical college now has its bacteriological laboratory. It is true that the course of bacteriology is not an uninterrupted march of attainment. This is shown in the present status of the Koch treatment of tubercular disease. Hardly more than a year has elapsed since what was announced as a triumph of the first magnitude came to us from the very vital knot of bacteriology; today it seems little better than a fiasco. Great as our disappointment is, is there not a wholesome lesson to be drawn from the fate of Koch's lymph? Should we not infer that closest research ought always to be checked by experience in real life; and would not such an inference lead to greater safeguards against loss of time and misdirection of energy in our quest after the secrets of nature? If we must own to defeat for the time being, surely our forces are not routed. Who doubts that tubercular disease, cancer, leprosy, and the acute infectious diseases will, before many years have passed, be made as tractable as those we now encounter with every expectation of victory, although our forefathers were powerless against them?

Preventive medicine, sanitation, hygiene—call it by whatever name you will—is walking hand in hand with bacteriology; and the two are at many points so blended as to be almost identical.

Disastrous epidemics there are still, no doubt, but none of the terrible destructiveness so common a century or two ago. This is because we do not now content ourselves with simply referring to an epidemic pestilence as being a "dispensation of Providence;" but seek by all possible means to find out the cause of infectious disease, and the methods of rendering those causes inoperative. We are constantly trying to get the mastery of pestilential diseases as Jenner mastered smallpox a little less than a century ago. That we shall succeed in time there is abundant reason to believe. I might mention many other achievements in medical science, either completed or in course of completion, that have aided powerfully in securing an evener distribution of human happiness than was possible in former times; but I will content myself with one—that of establishing the conviction in all civilized communities that insanity is not demoniacal possession, but the expression of disease.

Ever since Esquirol's time has it taken to do this; but it has been done. The insane are now the subjects of the utmost consideration, the tenderest solicitude, and the most painstaking care on the part of the individual and the state. Palatial hospitals and kindness have taken the place of the wretched abodes and the gross ill-treatment too often given them, even in comparatively recent times. We do not now punish the criminal insane—meaning those who commit acts that in others would be criminal; we confine them, to be sure, in their own interest and for the welfare of the community; but we manage them almost without even "chemical restraint." And I am glad to be able to truly allege that our own great State of New York is not surpassed by any State in the Union in thoughtful and generous treatment of the insane. I have thus mentioned a few of the more striking of the manifestations of progress in the pursuit of medicine.

To what extent have we in this part of the world—in this country, in the State of New York, and even in the County of Erie—contributed? Not to an overwhelming extent, it is true; still, bearing in mind the disadvantages under which we have labored, we need not feel that we have been backward in taking our part in the good work. It was in the United States that surgical anesthesia was first accomplished; that ovariotomy was first performed; and that litholapaxy was made to supplant lithotomy and lithotroty to a great degree. It was in the State of New York that chloroform was first discovered, that the elastic extension in treatment of fractures was first applied; and that college clinics were first held by Willard Parker. In New York, Bull has made many successful laparatomies for gunshot injuries of the intestines. In New York, Marion Sims, the epoch-making gynecologist, founded the first hospital devoted to the treatment of diseases and injuries peculiar to women. In New York, Sayre added greatly to our knowledge of the proper treatment of spinal curvature, and he has given us many new and valuable ideas in orthopedic surgery. In New York, Wood made known the powers of nature in the regeneration of bone. In New York, Clark taught and practised the opium treatment of peritonitis, though his extreme views have been antagonized by modern investigators. In New York, Stearns did invaluable work when he established confidence in the oxytocic power of ergot in the early days of its use. In New York, Otis revolutionized the diagnosis and treatment of urethral stricture. In New York, O'Dwyer perfected intubation of the larynx, and made it practicable.

These are but some of the striking achievements in medicine and surgery, that have happened in our own State. When we add to this list the familiar names of Thomas, Emmet, Barker, Taylor, Post, Sands, Carnochan, and many others whose achievements in their special fields of work have given them world-wide reputation, we have a list of names at once an honor to medical science and to America. As for our own County of Erie and our own City of Buffalo, we can fairly claim that our profession has kept well to the front. The County of Erie and the City of Buffalo gave to the medical world that illustrious trio, Flint, Hamilton and Dalton, the foundation of whose experience and fame was laid here. It was Erie County and Buffalo that furnished them the clinical material from which they obtained the knowledge out of which grew those published works which are looked upon as standard authorities in all lands. A little later the leading minds of our profession here were White, Rochester, Miner, Moore, (of Rochester, N. Y.,) Hadley, Lee, Eastman, Milton G. Potter, and the late lamented Burwell and Frank H. Potter; besides, others who could be named if time permitted. At the present time we have in our city many distinguished medical men, some of them of national repute. Prominent among these is Roswell Park, the first in this State to localize and operate successfully upon a cortical lesion of the brain, ante-dating Horsley in this field by about three months. To him also is due the credit of having been the first in America to successfully extirpate the larynx. His fame as a pathologist led the College of Physicians and Surgeons, of Philadelphia, to invite him to deliver the Mütter lectures for 1890-91, he being the first non-resident of Philadelphia to whom this great compliment has been extended. Another name is that of M. D. Mann, whose operative skill, together with his System of American Gynecology, a book which is considered one of the finest in the English language, entitles him to high rank among American gynecologists. Again, Herman Mynter, a successful teacher and practitioner of surgery, who has won credit for himself and his city through his surgical skill, as evidenced, for example, by his excellent record in his operations for the relief of perityphlitis. I feel that I should mention John Parmenter, who has done considerable and creditable work in the surgical field, and who recently and successfully performed the rare operation of open arthrotomy for subclavicular dislocation of the humerus,—the fourth operation of its kind that has been done in this country. Stockton, also, through the more extended use of gastric lavage, has done much towards changing our ideas of the therapy of stomach difficulties. And so I might go on, did time and space permit, with mention of men of recognized ability as teachers, and who are frequent and valued contributors to medical literature. Such for instance are W. W. Potter, J. B. Andrews, Cronyn, Lothrop, Howe, Abbott, Hopkins, Hubbell, Bartlett, Putnam, John Parmenter, W. C. Krauss, Hartwig, Pryor, Crego, John A. Miller, Cary, Wende, Fell, DeLancey Rochester, Hurd, Hayd and Grove.

Nor is our city lacking in opportunities for clinical study. In addition to the large and excellent corps of teachers connected with our two medical universities (both of them institutions of excellent reputation), we have three large general hospitals, namely : the Buffalo General Hospital, the Sisters' of Charity Hospital, and the Erie County Hospital, the combined capacity of these being about seven hundred beds. To these we may add the Fitch Accident Hospital and the Emergency Hospital, the ambulance service in both of which is unexcelled ; and to these we may add, again, several well-appointed and well-conducted dispensaries, representing the various special departments of medicine. The insane are well cared for in this city by the large and well-equipped State Hospital, and by the Providence Retreat, on Main Street, near Highland Park. The latter is conducted under the management of the Sisters of Charity, is delightfully located, and with its admirable staff of physicians, is one of the finest private hospitals for the insane in this State. Therefore, the student who seeks to obtain his medical education in Buffalo will most assuredly find that the opportunities afforded him will in every respect, be as good as those furnished by any city in the United States.

Retrospects of this kind are cheering; but, let us not copy the *gens d'armes* in the opera, who lose time in melodious self-congratulations while the fugitive, of whom they are in pursuit, diligently widens the distance between them and himself. It rests with us to press on constantly; only stopping to take in the situation, so to speak, to make sure that we are not on a false scent.

That in this honorable contest, the medical men of the County of Erie, the members of this Society, will always stand well forward in the ranks, I firmly believe; and you all believe it, for I am sure that you have resolved that it shall be so.

